# Editorial: Social Belongingness and Well-Being: International Perspectives

**DOI:** 10.3389/fpsyg.2021.735507

**Published:** 2021-08-30

**Authors:** Juan Carlos Oyanedel, Dario Paez

**Affiliations:** ^1^Facultad de Educación y Ciencias Sociales, Universidad Andres Bello, Santiago, Chile; ^2^Faculty of Psychology, University of the Basque Country, San Sebastian, Spain

**Keywords:** well–being, belongingness, social cohesion, social identity, social capital (culture), social integration, social support

This Research Topic presents a set of studies examining the psychosocial determinants of Well-Being (WB). They come from different parts of the world, and several of them are cross-cultural. Rather than merely reporting what they say, we believe this editorial can serve in a better way positioning them among the debate regarding the different concepts currently used to report this still nebulous field of belongingness.

The literature on social relations and WB describes several concepts as associated with social belongingness (SB). Among them, we can find social cohesion (SCo), social integration (SI), social support (SS), social capital (SC), social or group identification (IS), and belonging or relatedness needs (RN)—see Table 1.

**Table 1:** Concepts associated with Social Belongingness (SB).

**Construct**	**Relationship with SB**	**Definition**
Social cohesion	As identification and sense of attachment to different groups.	It represents the strength of social bonds and social equality within social networks.
Social integration	As participation in social networks and perceived connectedness.	It is a multidimensional construct that can be defined as the extent to which individuals participate in a variety of social relationships.
Social support	As a positive consequence of belongingness to a network of social support	Structural social support is the extent to which a person is connected within a social network. Subjective social support is the satisfactory reception and/or availability of different functional forms of social support.
Social capital	As social trust, reciprocity and altruism.	Networks together with shared norms, values and understandings that facilitate co-operation within or among groups. Access to resources through these networks.
Social identification	As identification, solidarity and satisfaction with different in-groups'.	It is the subjective dimension of social integration; namely, the extent to which individuals identify themselves as part of a given group.
Relatedness	As satisfaction of relatedness needs.	The quality or meaning of social connections. The extent a person feels personally accepted, respected, included, and supported by others in the social “environment.”
Social belongingness to institutions	As SB to an institution or an institutional framework	Feeling of belongingness or inclusion in an institution or institutional framework (to a set of institutions in the framework of a nation-state).

Durkheim defines SCo as a characteristic of a society that shows strong social bonds (Durkheim, 1897/1963). A recent conceptual review includes in SCo a social-psychological perspective of it as an attraction to the group and social capital (Schiefer and van der Noll, 2016). The definition of SCo from social psychology emphasizes the subjective aspect, affirming that it is the “degree of consensus felt by the members of a social group in the perception of belonging to a common project or situation” (Morales et al., 2007: 810). SCo could be defined as a collective attribute from a macrosocial approach, indicating the extent of connectedness and solidarity among groups in society. Social belongingness is an aspect or consequence of higher SCo, a subjective feeling of social integration related to identification and attachment to groups that enhances WB (Schiefer and van der Noll, 2016). Based on Schiefel and van der Noll, an SCo index was created, including social participation, the strength of social networks, social trust (people and institutions), respect for rules, solidarity—the first components are similar to SC (see Table 2 for items). Controlling GDP and Gini Index, the national mean of SCo predicts B = 0.17 individual WB in 34 nations (Dragolov et al., 2016).

**Table 2:** Constructs and measures related to Social Belongingness (SB).

**Construct**	**Measure**	**Examples**	**Meta-analytical correlations**
Social cohesion The degree of social bonds and social equality within social networks. SB as identification with different social groups.	Index of social Cohesion (Dragolov et al., 2016)	Items on social participation, strength of social networks, social trust (people and institutions), respect for rules, solidarity—giving social support, and national identification	Social cohesion correlates 0.61 with well-being—collective level
Social Integration extent to which individuals participate in a variety of social relationships. SB as connectedness	UCLA Loneliness Scale. (Russell, 1996)	Number of social contacts. Living alone or not married vs. the opposite. Social integration Participation in a broad range of social relationships. Perception of loneliness. Feelings of isolation, disconnectedness, and not belonging. (Holt-Lunstad et al., 2010, 2015).	Social integration correlates *r* = −0.12 and low loneliness *r* = −0.10 with mortality
Social Support Structural support extent to which a person is connected within a social network. Subjective social support: satisfactory reception and/or availability of different functional forms of social support. SB as positive consequences of social network	Questionnaire on the frequency of and satisfaction with social support (QFSSS). (Garcia and Rimé, 2019)	The frequency of and satisfaction with support received from your partner, family, friends, and the community. Emotional SS (e.g., “Your partner is loving, affectionate and listens to you when you want to talk and express your feelings”). Instrumental SS (e.g., “Would [a person] do you a favor if needed or is willing to do specific things for you, such as providing money, taking you to the doctor, or helping you in any other activity”). Informational SS (e.g., “[a person] Gives you useful advice and information regarding questions, problems, or daily tasks?”) (García-Martín et al., 2016).	Social support correlates *r* = −0.07 −0.12 with mortality and *r* = 0.17 −0.20 to well-being.
Social capital Size networks, bonding (in-group relations), or bridging (intergroup). Participation in organizations. SB as effect of social trust, reciprocity, and altruism.	Personal social capital (Wang et al., 2014). CSCS scale (Forsell et al., 2020).	Several dimensions including: Network size (e.g., “How do you rate the number of your friends?”); Trust (e.g., “Among your coworkers/fellows, how many you can trust?”); Resources (e.g., “Among all your relatives, neighbors, friends, co-workers, and classmates, how many have broad connections with others?”) and Reciprocity (e.g., “How many of your coworkers/fellows will definitely help you upon your request?”) (Wang et al., 2014). Norms of Behavior; Efficacy; Social Control (e.g., “[name organization] members behaving inappropriately are reprimanded”) and Reciprocity (e.g., “Members who help other members know the favor will be returned”) (Forsell et al., 2020).	Social capital correlates *r* = −0.043 with mortality and *r* = 0.066 with perceived health.
Social identification Subjective dimension of social integration; extent to which individuals identify themselves as part of a given group. SB as identification, solidarity and satisfaction with in-groups'	Multicomponent Model of In-group Identification	Self-definition dimension: the degree of Self-stereotyping (e.g., “I am similar to the typical/ average person of the in-group”), perceived In-group homogeneity (e.g., “In-group people are very similar to each other”). From a Self-investment dimension, the degree of perceived Solidarity (e.g., “I feel committed to [in-group]”), Positive Evaluation (e.g., “I am glad to be [in-group]”), or the Importance of the group to self or Centrality (e.g., “Being [in-group] is an important part of how I see myself”) (Leach et al., 2008).	Social identification correlates *r* = −0.15 with depression and *r* = 0.21 with well-being.
Relatedness	Basic needs satisfaction in general scale.	Item examples include: “I really like the people I interact with,” “I get along with people I come into contact with,” “I pretty much keep to myself and do not have a lot of social contacts” (reversely coded), or “I consider the people I regularly interact with to be my friends.” (Johnston and Finney, 2010).	Relatedness correlates *r* = 0.39 with positive affect.
Belongingness	The General Belongingness Scale (GBS).	Items examples include “When I am with other people, I feel included”; “I have close bonds with family and friends”; “I feel accepted by others; I have a sense of belonging”; “I have a place at the table with others” and “I feel connected with other” (Malone et al., 2012).	SB correlates *r* = 0.37 with well-being.

In this monograph, five studies deal with SCo and WB. Both Wlodarczyk et al. and Zumeta et al. show that effervescence during collective gatherings or perceived emotional synchrony is the primary mechanism explaining positive effects on social cohesion and WB of demonstrations and rituals (see Table 3). Reyes-Valenzuela et al. examined responses to collective trauma or disasters. They found that the intensity of the trauma influences social well-being through the mediation of collective effervescence or social sharing of emotions and community appraisal, allowing communities to cope collectively with extreme adverse events. Bravo et al. report that identification with the national football team predicts collective pride that mediates the relationship between identification with the national team and WB (see Table 3). Finally, Torres et al. report that the frontiers between individual well-being and community and even national well-being are diffuse, challenging the idea of subjective well-being only as an individually based phenomenon.

**Table 3:** Effects of participation in collective gatherings and perceived emotional synchrony on well-being.

**Consequences**	**Examples**	**Relation with SB and/or WB**
High positive affect and self-esteem.	Páez et al. (2015), Bouchat et al. (2020).	Increase Affect Balance in SWB and self-acceptance in PWB
Positive and self-transcendent emotions (e.g., joy, awe).	Pizarro et al. (2018).	Increases affect balance in SWB, mastery and purpose in life in PWB
Fusion of identity.	Páez et al. (2015).	Increases Positive relationships with others and purpose in life in PWB
Social integration.	Pelletier (2018).	Increases Positive affect in SWB, Positive relationships with others in PWB
Positive shared beliefs and social values.	Páez et al. (2015), Garcia and Rimé (2019).	Increases purpose in life and personal growth in PWB.

SC refers to a successful level of social integration that affords SB. Studies in SC tradition characterize social cohesion by strong social networks and a high level of generalized trust (Ponthieux, 2006). A meta-review concludes that nine reviews provided strong, and sixteen provided weak to moderate evidence that SC is related to health (Ehsan et al., 2019). Three meta-analyses found minor SC effects on mortality and self-rated health (Gilbert et al., 2013; Choi et al., 2014; Nyqvist et al., 2014). Some studies even suggest associations between the perception of health service treatment and subjective well-being (Rubio et al., 2020). Individuals with a more extensive social network, who perceived higher social cohesion and trusted their neighbors, were more likely to report higher WB (Hart et al., 2018). Montero et al., found that more segregated zones show higher WB, probably because neighborhoods' income homogeneity reinforces social capital. Da Costa et al. show that micro-level factors such as a transformational culture, close to a high SC in the organization, are directly and indirectly associated with individual WB through psychosocial factors like low stress, high role autonomy, social support, and quality leadership. These results are consistent with longitudinal work reported on the relationship between work and subjective well-being (Unanue et al., 2017). However, meso-social factors influence only social WB or emotional climate. Lopez et al. found that a positive school climate, beyond students' individual socio-demographic and family support, predicts WB. Labra et al. show that passing through the university increases the likelihood of forming friendship networks, a kind of social capital that can reduce socioeconomic segregation in highly unequal societies.

SI is defined as the frequency and number of social contacts or as the number of social ties or social network size. Durkheim-inspired SI theory posits that WB would be proportional to the degree of social integration of people in the groups they belong to (Berkman et al., 2000). Three meta-analyses found small associations between mortality and quantitative SI and subjective SI or low loneliness (Schwarzer and Leppin, 1989; Holt-Lunstad et al., 2010, 2015). Ventura-Leon et al. report that a scale of fear of loneliness correlates negatively with WB.

Another literature emphasizes the construct of SS as essential to the positive effect of SB. House et al. (1988) identify SS as a process through which networks or structures of social relations may influence WB. *Structural SS* refers to how a person is integrated within a social network, like the number of social ties (see social network item in SC). *Functional SS* looks at the specific functions that members in this social network can provide, such as emotional, instrumental, and informational support (House et al., 1988; Turner and Turner, 2013). In this sense, SB is related to satisfactory functional SS. Four meta-analyses (Schwarzer and Leppin, 1989; Pinquart and Sorensen, 2000; Chu et al., 2010; Bender et al., 2019) found that subjective and objective SS correlates with WB. Marenco-Escudero et al. found an association between SS and network size with community empowerment—but marginally significant. Quintero et al. found that the WB of victims of collective violence in Colombia who returned to their places of origin was higher than those who chose to settle elsewhere, probably because the first could strengthen SS in their old neighborhood's networks. Cobo-Rendon et al. show that people who improve their balance of affects increase SS, while those who worsen it decrease it. Donoso et al. examine the effects of social media on well-being, reporting that intense use of the internet for social, recreational, and educational purposes, as long as it is not problematic, has a positive association with students' subjective well-being.

Social psychologists posit that the current operationalization of social integration as the frequency of social contacts neglects the subjective dimension, namely IS (Postmes et al., 2018). Leach et al. (2008) proposed the Group Identification Scale. This scale includes a self-definition dimension, focusing on the perceived similarity of the self to prototypical members of the in-group. This dimension also includes an appraisal of the in-group homogeneity. The scale also has a self-investment dimension, referring to importance, solidarity (“I feel committed to [in-group]”), and satisfaction (“I am glad to be [in-group]”) (Leach et al., 2008). The last two components of identification are akin to SB. WB correlates with organizational IS (Steffens et al., 2016) and ethnic IS (Smith and Silva, 2011). IS was found to be negatively associated with depression (Postmes et al., 2018). Some studies found that IS predicts WB better than quantitative social support (Sani et al., 2012), while other studies found that SS was a strong predictor than IS of WB (Haslam et al., 2005). Zabala et al. support the association between Basque IS, collective empowerment, and WB. Cuadros et al. show structural validity of collective esteem scale, and scale scores correlate with WB. Pinto et al. find that the European supranational identity is associated with social WB or a climate of prosocial behavior and migrant inclusion. Garcia et al. show that ethnic IS buffers distress in the face of discrimination and is associated with WB. Moyano-Díaz and Mendoza-Llanos show that participation in groups with a sense of belonging to the neighborhood, such as community-based organizations, is associated with WB. Nonetheless, it seems to be social identification with the neighborhood -and not belongingness- that predicts WB. Navarro-Carrillo et al. analyze a classic issue: how objective and subjective measures of socioeconomic status correlate with WB – *r* = 0.16 and *r* = 0.22, respectively (Zell and Strickhouser, 2018). They adapted the MacArthur pictorial social ladder to income, education, and occupation that emerged as predictors of psychological WB over and above the MacArthur Scale.

Baumeister and Leary (1995) argue that belongingness is a social need, whose essential components are regular social contact and feelings of connectedness, and satisfaction of this need reinforces WB. Ryan and Deci (2017) postulates relatedness as a basic need that involves feeling a sense of support and connection with others and is akin to SB. The Basic Needs Satisfaction Scale measures relatedness with items like “I really like the people I interact with” (Johnston and Finney, 2010). A meta-analysis found that satisfaction of relatedness correlates with WB (Stanley et al., 2020). Pardede et al. explore different dimensions of belongingness. They do so by analyzing several items representing the need for acceptance and belongingness. They found only three correlated dimensions, a factor of belonging, one of emotional expression, and another of self-presentation to others—showing the importance of sharing emotions for SB. Gonzalez et al. found that subjective evaluation and functioning or satisfaction of basic needs, social ties, and respect predicts WB. They also report that functioning respect and human security predicts social WB. García-Cid et al. show that a sense of community that includes emotional connection with the group buffers the adverse effects of discrimination on the psychological WB of migrants. Urzua et al. examines the effect of discrimination in migrants and found that negative affect mediates between the first and low WB. Simkin examines the experiences of Latin American Jews that migrated to Israel and found that the centrality of this event was positively related to WB—probably because migration satisfies needs for belongingness and is congruent with self-transcendent beliefs. Oriol et al. analyze the role of self-transcendent prosocial aspirations, confirming that they were related directly to WB and indirectly through the self-transcendent emotion of gratitude.

Finally, a more focused tradition analyzes the association between social belonging and adjustment occurring in formal institutions, such as schools (Malone et al., 2012). Students who feel personally accepted and supported by others in the school report positive WB (Korpershoek et al., 2019). Paricio et al. found that group identification with the school correlates with hedonic and psychological WB. Mera-Lemp et al. show that self-efficacy reduced the negative effect of prejudice in satisfaction with school in immigrant students, suggesting that improving intercultural skills can increase SB in school. Cespedes et al. found that migrants students report a higher self-academic concept than native, but not higher WB. Correlations between academic self-concept and WB were lower in migrants than natives, showing the limits of academic success to enhance WB. Gempp and Gonzalez-Carrasco examine peer relatedness, school satisfaction, and life satisfaction in secondary school students. A reciprocal influence between school satisfaction and overall life satisfaction was found, and the association of peer relatedness with life satisfaction was fully mediated by school satisfaction.

## A Brief Balance and Future Challenges

The COVID 19 pandemic has meant fundamental challenges to the way we understand our nexuses with other people. Extended periods of isolation have affected the way we interact with people, replacing, for a high number of people, the usual face-to-face contact for a technologically mediated one. The pandemic has also limited the way we interact in face-to-face settings by enforcing social distancing rules. The need to be careful about the people around us has brought a new meaning to the Sartrean idea that “*L'enfer, c'est les autres*.”

These new ways of interacting have challenged several aspects of the way we used to create society. The pandemic has put a heavy strain on families, increasing their interactions in restricted spaces and sharing personal space for more extended and intense periods. It has challenged the way we educate, forcing the closure of schools and re-engaging the family in educational activities. It has also meant transformations in the world of work, which most probably will have a long-lasting effect, such as remote working agreements. These changes will affect traditional sources of socialization, such as labor unions or educational institutions, requiring them to update and adapt to this new, less territorially based world or witnessing a severe weakening of their social relevance. Finally, it has also affected the way we celebrate and play, modifying the way collective gatherings and rituals are enacted.

While we develop a “psychosocial vaccine” to prevent the long-lasting adverse psychological effects of the pandemic, we must remember that what makes us human is, at the very end, to be among humans.

We expect that the articles contained in this Research Topic have contributed to this end, highlighting the complexities of researching how belongingness affects our well-being.

## Author Contributions

All authors listed have made a substantial, direct and intellectual contribution to the work, and approved it for publication.

## Conflict of Interest

The authors declare that the research was conducted in the absence of any commercial or financial relationships that could be construed as a potential conflict of interest.

## Publisher's Note

All claims expressed in this article are solely those of the authors and do not necessarily represent those of their affiliated organizations, or those of the publisher, the editors and the reviewers. Any product that may be evaluated in this article, or claim that may be made by its manufacturer, is not guaranteed or endorsed by the publisher.
